# Pregnancy eating attributes study (PEAS): a cohort study examining behavioral and environmental influences on diet and weight change in pregnancy and postpartum

**DOI:** 10.1186/s40795-016-0083-5

**Published:** 2016-07-15

**Authors:** Tonja R. Nansel, Leah M. Lipsky, Anna Maria Siega-Riz, Kyle Burger, Myles Faith, Aiyi Liu

**Affiliations:** 1 Health Behavior Branch, Division of Intramural Population Health Research, Eunice Kennedy Shriver National Institute of Child Health and Human Development, 6710B Rockledge Dr., MSC 7004, Bethesda, MD 20892, USA; 2 Gillings School of Global Public Health, University of North Carolina Chapel Hill, 2105-A McGavran-Greenberg Hall, CB 7435, Chapel Hill, NC 27599, USA; 3 Gillings School of Global Public Health, University of North Carolina Chapel Hill, 2204 McGavran-Greenberg Hall, CB# 7461, Chapel Hill, NC 27599-7461, USA; 4 Department of Counseling, School, and Educational Psychology, Graduate School of Education, University at Buffalo – SUNY, Buffalo, NY 14250-1000, USA; 5 Biostatistics and Bioinformatics Branch, Division of Intramural Population Health Research, Eunice Kennedy Shriver National Institute of Child Health and Human Development, 6710B Rockledge Dr., MSC 7004, Bethesda, MD 20892, USA

**Keywords:** Diet, Pregnancy, Food reward sensitivity, Eating behavior, Gestational weight gain, Postpartum weight retention

## Abstract

**Background:**

The rising prevalence of maternal overweight/obesity and excessive gestational weight gain poses a serious public health concern due to the contribution of these factors to increased risk of negative health outcomes for both mother and child. Scant intervention research has indicated moderate short-term improvement in maternal diet and gestational weight gain, with little evidence of long-term behavior change, in parallel with findings from interventions outside of pregnancy. Recent laboratory-based findings from neuroscience implicate aberrant reward processing of food at the brain level (“food reward sensitivity,” the between-individual variation in the response to food stimuli) as a contributor to eating beyond energy needs. However, scant research has examined the influence of these processes on weight change in population-based settings, and the relevance of these processes to pregnancy-related weight change has not been explored. The purpose of the Pregnancy Eating Attributes Study (PEAS) is to examine the role of food reward sensitivity in maternal diet and weight change during pregnancy and postpartum. The study examines the interplay of food reward sensitivity with behavioral control, home food environment, and related aspects of eating behavior in the context of weight-related biomedical, psychosocial, genetic and behavioral factors including physical activity, stress, sleep and depression.

**Methods:**

Women of varying baseline weight status (n = 450) are enrolled early in pregnancy and followed, along with their infants, until 1 year postpartum. Assessments occur during each trimester of pregnancy, and postpartum at approximately 2 months, 6 months, 9 months and 12 months. Maternal food reward, self-control, home food environment, eating behaviors, dietary intake, health behaviors, and anthropometrics are assessed along with maternal and infant clinical and biological data, infant anthropometrics, and feeding practices. Primary exposures of interest include food reward sensitivity, behavioral control, and home food environment. Primary outcomes include gestational weight gain, postpartum weight retention and maternal diet quality.

**Discussion:**

With increasing evidence suggesting the relevance of food reward sensitivity for understanding eating behavior, PEAS aims to advance understanding of the determinants of eating behavior during pregnancy, informing future interventions for improving maternal diet and weight change, and leading to improved maternal and child health and weight trajectories.

**Trial registration:**

Clinicaltrials.gov, NCT02217462. Date of registration: August 13, 2014

## Background

Approximately two-thirds of women of reproductive age are overweight or obese [1], and across the range of body mass index (BMI), gestational weight gain (GWG) in excess of guidelines is more common than GWG within or below guidelines, contributing to maternal obesity risk, pregnancy complications, and unsuccessful breastfeeding [1]. Adverse infant outcomes associated with maternal obesity and excessive GWG include birth defects, macrosomia, shoulder dystocia, perinatal mortality, hyperinsulinemia, developmental delays, childhood obesity and cardiovascular disease [2–15]. Maternal diet is increasingly recognized as an important factor in the developmental origins of health and disease. Antecedents of obesity may develop in utero [16, 17], and data suggest that altering maternal prenatal diet impacts off-spring body composition [18–23] as well as a range of adverse child outcomes including birth defects [24], cancer [25–28], type 1 diabetes [29], and asthma symptoms [30]. Research is needed to identify dietary determinants of excessive GWG and postpartum weight retention, and inform best practices for weight management during pregnancy and the postpartum period [31].

The problem of obesity and weight gain in pregnancy is linked to the larger epidemic of obesity in the US which, along with poor diet quality, contributes to numerous adverse health outcomes, including reduced fecundity and fertility and chronic diseases such as cardiovascular disease, sleep disorders, and many cancers [32]. The poor diet quality of the US population, characterized by excessive intake of total energy, added sugar, fat and sodium, and inadequate intake of fruit, vegetables and whole grains, is well-documented [33– 37]. Mirroring findings regarding weight management in pregnancy, weight management interventions in the general population have been only marginally successful, with less than optimal initial weight loss and poor long-term maintenance [38–41].

The most proximal cause for excess body weight is eating beyond energetic needs. An emerging hypothesis for widespread excess energy intake leading to weight gain and increased adiposity is excess “hedonically motivated food intake” [42], which posits that the neural reward response to highly palatable food cues acts to override the homeostatic processes that historically balanced energy intake with energy expenditure, e.g., [42, 43]. Human neuroimaging research has revealed differential mesolimbic/mesocortical reward circuitry response to both consuming and viewing images of highly palatable foods versus control stimuli (e.g., non-food objects and tasteless solution) as a function of weight status [44]. Differences in reward-related brain activity in the nucleus accumbens in response to food images predicted weight change across 6 months among college students [45]. These neuroimaging results dovetail with research using behavioral assessments of food reinforcement value – the degree to which individuals are willing to work for food rewards – which also implicates the reinforcing value of food as an important determinant of dietary intake and weight status [46].

Evidence suggests that the neural reward response to food stimuli is dependent on a number of food attributes, indicating that highly palatable foods may be more likely than others to contribute to hedonically-driven overeating. Neural reward response is positively associated with perceived energy content of food in images [47–49], and as such, foods high in reinforcement value are generally high-fat, high-sugar, high-sodium selections [50–55]. It is likely, then, that foods processed to contain added fat, sugar, and salt including desserts/ pastries, candy, sweetened cereal, snack chips, cheese, fried foods, and processed meat – all highly prevalent in the US diet [56, 57] – are high in reinforcement value. In comparison, nutrient-dense foods containing minimal added sugar, fat and sodium such as whole grains, vegetables, fruit and legumes [58, 59] may be less likely to result in hedonic overeating, and are referred to herein as “normo-palatable” foods. Relative to their highly palatable counterparts, images of these normo-palatable foods elicit a lower reward and attentional response [48, 60–62]. Consequently, the pervasive availability, accessibility and low cost of highly palatable foods may displace normo-palatable foods in the diet [63], leading to excessive intake of energy, sugar, fat, and sodium, inadequate intake of nutrient-dense foods, and excess weight gain.

Basic, behavioral, and neuroimaging research has identified a number of important factors associated with inter-individual differences in the neural reward response to food stimuli and its plasticity. Repeated over-eating of highly palatable foods is hypothesized to be a function of individuals’ amplified reward response to highly palatable foods and/or associated food cues [64– 67]. Studies have demonstrated the reward response to cues for energy dense food is more pronounced in obese versus normal weight subjects [62], and predicts subsequent weight gain over 6–12 months [61]. Experiments that randomly assigned young adults to consume high-fat/high-sugar foods daily over 14–22 days showed increased food reinforcement for their assigned food relative to controls [68, 69], echoing findings with rodents [70]. Such findings provide compelling behavioral evidence that “wanting” for highly rewarding foods increases with repeated intake. Also in line with the neuroplasticity hypotheses, sensitivity to specific highly palatable foods has been shown to decrease over time in subjects frequently consuming these items [71]. Subjects assigned to consume high-fat/high-sugar foods daily over 14–22 days [72–74] or even 3-month periods [75] reported reduced “liking” of the foods relative to baseline and control foods that were not consumed daily. Taken together, laboratory and experimental evidence from non-pregnant samples implicate the importance of both food-specific attributes and between-individual differences in the role of the neural reward response to food stimuli on dietary intake and weight change. However, no study to date has evaluated these constructs in the context of maternal weight change during pregnancy and postpartum.

In addition to the contributions of reward responsivity and food reinforcement value to eating behavior, evidence also indicates that decreased self-regulation may be a risk factor for overeating and excess weight gain. Both self-report and laboratory measures of impulsivity correlate positively with caloric intake [76, 77] and body mass index [78–80]. Two studies demonstrated that the influence of food reward sensitivity on intake is modified by behavioral control. Findings from both studies indicate that food reward sensitivity is positively associated with food intake during an “eating in the absence of hunger” research paradigm (EAH) particularly in the presence of high versus low impulsivity [81, 82]. Dietary restraint, defined as actively attempting to control intake in order to produce weight loss or prevent weight gain, has also been positively associated with food reward sensitivity [64], and has been found to moderate the effect of food reward on adult energy intake and body weight [83]. Restraint was inversely related to snack food intake in one cross-sectional observational study of college students with normal weight status, but was positively related to snack food intake in overweight subjects [84]. This suggests a distinction between “successful” and “unsuccessful” restrainers, potentially due to differences in self-regulation skills as well as food reward sensitivity [85]. Women of normal, overweight, or obese weight status who demonstrated restrained eating behaviors prior to pregnancy were more likely to experience excessive weight gain [86], possibly suggesting that efforts to control eating were relaxed during pregnancy. Recent evidence indicates that low impulsivity is associated with reduced functional connectivity between the reward and affect regions of the brain, a process involved in cost/ benefit evaluation of primary rewards [87]. Further, neural response associated with greater generalized and food-specific impulsivity is related to elevated BMI and predicts future weight gain [48, 88–90]. Despite this growing body of evidence in non-pregnant populations, no study to date has examined the role of food reward and its interaction with behavioral control in the context of GWG and postpartum weight change.

Previous interventions to improve dietary intake and promote healthy body weight based on current health behavior theories have yielded minimal success, suggesting the need for a more comprehensive understanding of the determinants of eating behavior. The reward-driven motivation to eat, culminating in widespread susceptibility to the influence of high food reinforcement value (“hyper-palatability”) on overeating, may be a central factor that has been absent from interventions to influence eating behaviors and weight change. Progress in understanding determinants of GWG and postpartum weight change may be advanced by extending recent findings from lab-based research on the neural reward response to highly palatable food cues to large-scale population-based research. Unique aspects of pregnancy further support the scientific utility of investigating the role of these constructs in diet and weight change. The period of pregnancy and postpartum is a bounded time of expected weight change, offering a window during which influences on weight change may be investigated. An increase in dietary intake is socially normative (e.g., beliefs regarding “eating for two”), and consequently, efforts to restrain intake may be relaxed. Additionally, because many women do not return to their pre-pregnancy weight, excess GWG and postpartum weight retention represent important risk factors for long-term excess weight. The proposed research investigates the influence of food reward sensitivity, home food environment, and behavioral control on maternal dietary intake and weight outcomes during pregnancy and postpartum. The study will further explore these constructs in the context of other aspects of eating behavior such as dietary restraint and motivation for healthful eating, as well as weight-related biomedical, psychosocial and behavioral factors including genetics, physical activity, stress, sleep and depression.

### Conceptual model

**Fig. 1:**
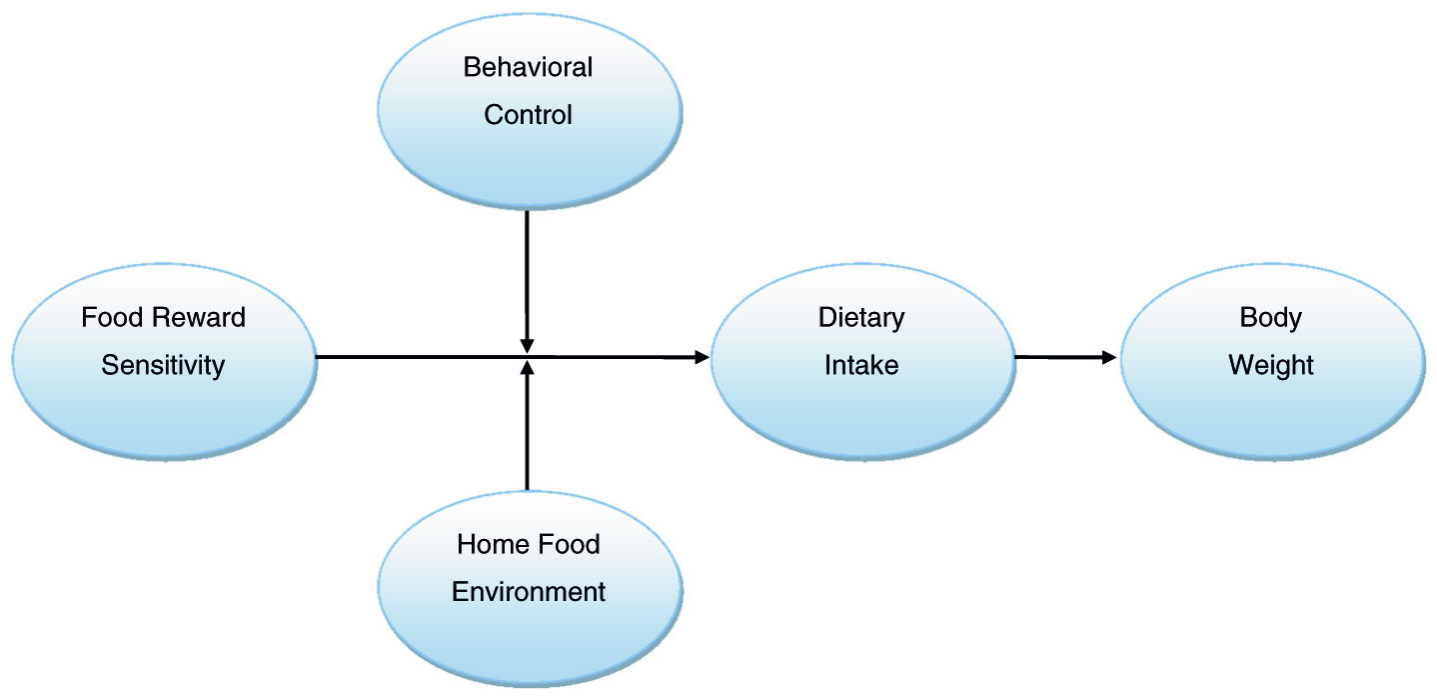
Pregnancy Eating Attributes Study (PEAS) conceptual model

The hypothesized core conceptual model (Fig. 1) underlying this research considers the primary constructs to be examined in this study with regard to their hypothesized influence on eating behavior and weight change.

Food reward sensitivity is a within-person characteristic posited to influence dietary intake and body weight outcomes. All else held constant, individuals with higher food reward sensitivity are hypothesized to consume more food, leading to excess energy intake and increased risk of excessive weight gain and overweight/obesity.

Behavioral control (self-regulation) is the other key personal factor influencing dietary intake and weight outcomes [81–83]. Individuals with high food reward sensitivity but sufficiently high behavioral control of intake may not consume excessively, whereas excess intake would be more likely for individuals with high food reward sensitivity and low self-regulation.

The primary environmental factor included in this model is the reinforcement value of foods in the home. The degree to which food environments are predominated by foods sufficiently high in reinforcement value so as to induce hedonic overeating is hypothesized to influence the relationship between food reward sensitivity, behavioral control, dietary intake and weight outcomes [83]. Individuals with high food reward sensitivity and low behavioral control who are exposed to mainly highly palatable food in their environment are hypothesized to be at increased risk for excess energy intake and elevated weight status. Conversely, even individuals with high food reward sensitivity and low behavioral control may be less like to experience excessive energy intake and weight gain if the home food environment contains few or no highly palatable foods.

More distal influences assessed in this study but not included in the conceptual model include personal factors such as stress, sleep and depression, which are hypothesized to influence food reward sensitivity, behavioral control and dietary intake directly. Consequently, these factors also impact dietary intake and weight outcomes through indirect pathways. Physical activity is hypothesized to influence energy expenditure and weight outcomes directly. Genes are hypothesized to have direct effects on all interpersonal model components.

### Study aims

The overarching aim of this research is to examine the role of food reward sensitivity, food reinforcement value, and behavioral control in maternal weight change and dietary intake during pregnancy and postpartum. Primary research questions include the association of food reward sensitivity with maternal dietary intake and weight outcomes; the moderating role of behavioral control and the availability of high-reinforcement value foods in the home environment; and differences in food reinforcement value of fruits and vegetables in their natural form versus highly processed sweet and savory snacks. Secondary research questions include examination of the interplay of food reward sensitivity, self-control, and the home food environment with other eating-related behaviors; the role of maternal food reward sensitivity and dietary intake on infant feeding behavior and body composition, and the roles of maternal sleep, stress and depression as potential moderators of the effect of food reward sensitivity on dietary intake, and change in body weight and body composition.

## Design and methods

### Study design

PEAS is a prospective observational study of 450 women without evidence of psychiatric or eating disorders, recruited in early pregnancy (≤12 weeks gestation), including those of normal weight (BMI 18.5–24.9 kg/m^2^), overweight (BMI 25–29.9 kg/m^2^) and obese (BMI ≥30 kg/m^2^). Women are followed through pregnancy and until 1-year postpartum, along with their infants from birth to 1 year, with collection of anthropometrics, blood, stool, and urine specimens, previous and current medical and demographic information, and dietary intake and eating and physical activity behaviors. We anticipated an 11 % attrition rate from enrollment to delivery and another 12 % attrition rate from delivery to 1 year postpartum for a total sample size of approximately 350 women-child dyads.

### Participants

Participants are recruited from women obtaining prenatal care at the obstetrics clinics at the University of North Carolina (UNC) at Chapel Hill Healthcare System with two locations, one in the hospital and the other off campus – Timberlyne. Inclusion criteria include the following: confirmed pregnant ≤12 weeks gestation at enrollment; uncomplicated singleton pregnancy anticipated; age ≥18 and <45 at screening; willingness to undergo study procedures and provide informed consent for her participation and assent for the baby’s participation; BMI ≥18.5 kg/m^2^; able to complete self-report assessments in English; access to Internet with email; plan to deliver at the UNC Women’s Hospital; and plan to remain in the geographical vicinity of the clinical site for 1 year following delivery. Exclusion criteria include the following: pre-existing diabetes (type 1 or type 2); multiple pregnancy; participant-reported eating disorder; any fetal anomaly requiring surgery with hospital admission following delivery (e.g., neural tube defects, gastroschisis, cardiac defects, Trisomy 21); any medical condition contraindicating participation in the study such as chronic illnesses or use of medication that could affect diet or weight (e.g., cancer, HIV, active renal disease, myocardial infarction in the last 6 months, chronic steroid use, thyroid disease requiring medication, or autoimmune disease such as rheumatoid arthritis, lupus, antiphospholipid antibody syndrome, scleroderma); psychosocial condition contraindicating participation in the study (e.g., bipolar disorder, schizophrenia, major affective disorder, and substance abuse). Recruitment of participants was initiated November 2014 and expected to be completed by December 2016.

### Procedures

Potential participants are identified through the electronic clinical appointments and medical records database. These women are approached regarding the study by research staff and provided with information regarding study participation, including referral to information on the study website. All participants provide signed informed consent to participate in the study. After delivery, participants additionally provide signed informed consent for their child’s participation. Follow-up comprises designated data collection at scheduled clinic visits for the mother during pregnancy and postpartum, and for the baby after delivery, as well as self-report assessments that the participant completes online by secure connection at the study website. Certain specified medical history, medication use, laboratory data and pregnancy complications are extracted from the electronic medical record system post-delivery. Study procedures were approved by the UNC Institutional Review Board.

### Measures

Study assessments are conducted prenatally at each trimester of pregnancy, and postpartum at approximately 2 months, 6 months, 9 months and 12 months. A summary of the data collection schedule is provided in Table 1.

Outcome measures include maternal anthropometrics (height and weight; waist, hip and mid upper arm circumferences; and triceps, iliac crest, and thigh skin folds), maternal dietary intake assessed using 24-hour dietary recalls [91, 92], infant eating behavior measures [93–96], and infant anthropometry (birthweight, weight, length, head/abdominal/mid-arm circumferences, skin-folds). Research staff are trained and certified on all these measures prior to formal data collection.

Individual’s food reward sensitivity is measured using several validated questionnaires [85, 97–99]. Reinforcement values of a variety of foods are assessed using adaptations to existing measures [100, 101]. The psychometrics of the measures will be evaluated. Related eating constructs will be assessed including restrained, external and emotional eating [102], motivation for healthy eating [103], eating competence [104], cravings and aversions (developed by the investigators for this study), and food preferences [105].

Self-regulation is measured using two validated questionnaires [106, 107]. The presence of foods with high reinforcement value in the home environment will be assessed using the Home Food Inventory [108], which yields an obesogenic home food availability score. Additional assessments include maternal weight history [109], physical activity [110], perceived stress [111, 112], sleep quality [113], nausea/vomiting, provider advice regarding GWG, and postpartum depression [114]. Data on participant obstetric history, health status, medication use, genetic screening, pregnancy progression (including lab and ultrasound data), and pregnancy complications are extracted from the electronic medical record. Biospecimens collected include maternal blood, urine, rectal swab, cord blood and child rectal swab. Participant demographic information including education level, family income, household composition, marital status, and race/ethnicity are obtained through maternal self-report.

### Power analyses

Power analyses are based on examination of the association of food reward sensitivity with GWG, energy intake, and diet quality. For analysis of GWG, the null hypothesis to be tested is that a subject’s weight gain during pregnancy is independent of the subject’s food reward sensitivity, using a regression model with weight gain as the dependent variable and food reward sensitivity score as the independent variable. The null hypothesis is then the regression coefficient beta in the model is zero. For the power calculation, we assume a mean food reward sensitivity score of 2.28 with standard deviation of 0.76, an overall mean GWG of 29.7 pounds with standard deviation of 11.7, both measured at three time points during pregnancy, and utilizing the average of the three measurements. Further assuming a correlation between two food reward sensitivity measurements of 0.7, with a standard deviation of the average score of 0.68, taking retention into account, and an effective sample size of 400 women, with a two-sided significance level of 0.05, the power will be at least 90 % to detect a regression coefficient of 2.76, interpreted as the estimated gain in gestational weight for every unit increase in the food reward sensitivity score.

For analysis of energy intake, the null hypothesis to be tested is that a subject’s energy intake is independent of the subject’s food reward sensitivity, using a regression model with energy intake as the dependent variable and food reward sensitivity as the independent variable. The null hypothesis is then the regression coefficient beta in the model is zero. Assuming energy intake has a mean of 2296 kcal and standard deviation of 453 kcal, and an effective sample size of 400, with a two-sided significance level of 0.05, the power will be at least 90 % to detect a regression coefficient of 106.83 kcal, interpreted as the estimated increase of dietary intake for every unit increase in the food reward sensitivity score.

The Healthy Eating Index-2010 (HEI-2010) is the primary indicator of diet quality. The HEI2010 score measures conformance to the 2010 Dietary Guidelines for Americans, and is comprised of 12 component scores corresponding to dietary guidelines for intake of total fruit, whole fruit, total vegetables, greens and beans, whole grains, dairy, total protein foods, seafood and plant proteins, fatty acids, refined grains, sodium, and empty calories [115]. The null hypothesis to be tested is that a subject’s HEI-2010 score is independent of the subject’s food reward sensitivity, using a regression model with HEI-20101 as the dependent variable and food reward sensitivity as the independent variable. The null hypothesis tests whether the regression coefficient beta in the model is zero. Assuming a mean HEI-2010 score of 52.7 with a standard deviation of 43.2, and a two-sided significance level of 0.05, the power will be at least 90 % to detect a regression coefficient of 10.21, interpreted as the estimated increase of HEI-2010 score with every unit increase in food reward sensitivity score.

### Substudies

Three substudies are embedded within the PEAS observational cohort study in order to examine food reward sensitivity and related constructs in greater depth than could be conducted in the main study. Substudies include focus groups, an experimental study using an eating in the absence of hunger paradigm, and functional magnetic resonance imaging (fMRI) of neural response to food stimuli.

### Focus groups

A series of focus groups is conducted to provide indepth exploration of participants’ perceptions relevant to food reward value and other influences on eating during pregnancy. Participants are recruited from the main cohort, including participants of normal weight, overweight, and obese weight categories, for a total of approximately 80 women. Inclusion/exclusion criteria are the same as those used for the main study.

### Eating in the absence of hunger

This substudy involves a behavioral experiment that investigates hedonic eating using an eating in the absence of hunger (EAH) paradigm. Aims of the substudy include examining the relationship of food reward sensitivity and BMI with EAH and the potential modifying role of self-control. Participants are recruited from the main cohort, to include participants from the normal weight, overweight, and obese weight categories, for a total of approximately 50 women. Women participate in the behavioral experiment at any point during the window of their second trimester. Inclusion/exclusion criteria are the same as those used for the main study. Additionally, eligible women must have no allergies or aversions to foods served in the substudy protocol.

### Functional neuroimaging

This substudy will evaluate brain response to multiple types of food stimuli, varied on hedonic value, as well as an examination of possible weight related differences in resting state functional connectivity and brain network organization using functional magnetic resonance imaging (fMRI). Specifically, this substudy will examine the relationship of brain response to food stimuli (e.g., cue-elicited anticipation and during receipt) with current weight status and GWG, test for the ability of brain response to food stimuli to predict postpartum weight retention (prospectively), evaluate between-food differences in brain response to food stimuli, as well as test for associations of brain response to food stimuli with survey and behavioral measures of food reward sensitivity. Approximately 75 participants, including normal weight, over-weight, and obese women, are recruited from the PEAS cohort to participate at their six-month postpartum study visit and undergo behavioral and survey assessments of food reward, executive functioning and one fMRI session.

## Discussion

The problem of maternal overweight, obesity, and excessive GWG is a critical public health concern. Findings from basic research in animals and humans indicate the reward-driven motivation to eat culminating in wide-spread susceptibility to the influence of highly palatable foods on overeating may be a central factor that is absent from current predominant theoretical frameworks explaining eating behavior. Research on the neurobiology of eating behavior has raised important unanswered questions that must be addressed in order to enable the translation to population-based research. The degree to which food reward sensitivity is associated with dietary intake and weight change during pregnancy is not well-understood, nor is the interplay of food reward sensitivity with other relevant constructs including self-control, the food environment, and other eating and health-related behaviors. This observational study addresses important knowledge gaps by examining the implications of food reward sensitivity for maternal diet and weight change. PEAS aims to advance understanding of the determinants of eating behavior and weight change, informing future interventions for improving maternal diet and obesity risk, and leading to improved maternal and child health trajectories.

## Figures and Tables

**Table 1:** Pregnancy Eating Attributes Study (PEAS) data collection and schedule

	Pregnancy			Delivery	Postpartum			
	1-15 weeks^a^	16-27 weeks	28-36 weeks		4–14 weeks	23–31 weeks	37–41 weeks	50–58 weeks
Dietary Intake								
24-hour Dietary Recall [91, 92]	X	X	X		X	X		
Food Reward								
Power of Food Scale [97, 98]	X	X	X			X		
Cravings & Aversions	X	X						X
Modified Yale Food Addiction Scale [99]	X					X		
Food Ratings [105]	X	X				X		
Food Reinforcement Questionnaire [85]	X	X				X		X
Multiple Choice Procedure [101]	X	X				X		
Behavioral Control								
Delaying Gratification Inventory [106]		X				X		
Barratt Impulsivity Scale [107]		X				X		
Other Eating Behaviors								
Dutch Eating Behavior Questionnaire [102]		X				X		X
Treatment Self -Regulation Questionnaire [103]	X					X		
Eating Competence [104]		X				X		
Food Environment								
Home Food Inventory [108]		X				X		
Infant Dietary Intake and Eating/Feeding Behaviors								
Breastfeeding intention		X						
Infant Food Intake and Breastfeeding [93, 94]					X	X	X	X
Baby Eating Behavior Questionnaire [95]								X
Feeding to Soothe [96]					X	X		X
Additional Health Behaviors								
Physical Activity Questionnaire [110]	X	X			X	X		X
Perceived Stress Scale [111, 112]		X	X		X	X		
Pittsburgh Sleep Quality Index [113]		X	X		X	X		
Edinburgh Postnatal Depression Scale [114]	X				X	X		
Weight History [109]	X							
Biomedical Data								
Maternal Anthropometrics	X	X	X		X	X		X
Infant Anthropometrics					X	X		X
Maternal Clinical Profile	X	X	X		X			X
Infant Clinical Profile					X	X		X
Maternal Blood^b^	X	X	X					X
Maternal Urine (first morning)		X						X
Maternal rectal swab		X						X
Cord Blood				X				
Infant rectal swab						X		

a The first clinic visit occurs at <12 weeks; self-report measures are completed by week 15

b 1^st^ and 3^rd^ collection are random; 2^nd^ and 4^th^ are fasting

## Abbreviations

BMI: body mass index
EAH: eating in the absence of hunger
fMRI: functional magnetic resonance imaging
GWG: gestational weight gain
HEI-2010: healthy eating index-2010
PEAS: pregnancy eating attributes study
UNC: University of North Carolina

## References

[1] Rasmussen KM, Yaktine AL, Institute of Medicine (US) and National Research Council (US) Committee to Reexamine IOM Pregnancy Weight Guidelines (2009). Weight gain during pregnancy: reexamining the guidelines.

[2] Baeten JM, Bukusi EA, Lambe M (2001). Pregnancy complications and outcomes among overweight and obese nulliparous women. Am J Public Health.

[3] Bodner K, Wierrani F, Grunberger W, Bodner-Adler B (2011). Influence of the mode of delivery on maternal and neonatal outcomes: a comparison between elective cesarean section and planned vaginal delivery in a low-risk obstetric population. Arch Gynecol Obstet.

[4] Callaway LK, Prins JB, Chang AM, McIntyre HD (2006). The prevalence and impact of overweight and obesity in an Australian obstetric population. Med J Aust.

[5] Castro LC, Avina RL (2002). Maternal obesity and pregnancy outcomes. Curr Opin Obstet Gynecol.

[6] Catalano PMaE HM (2006). The Short- and Long-Term Implications of Maternal Obesity on the Mother and Her Offspring. BJOG.

[7] Catalano PM, Presley L, Minium J, Mouzon SH (2009). Fetuses of Obese Mothers Develop Insulin Resistance in Utero. Diabetes Care.

[8] Catalano PM (2010). Obesity, Insulin Resistance, and Pregnancy Outcome. Reproduction.

[9] Chu SY, Callaghan WM, Kim SY, Schmid CH, Lau J, England LJ, Dietz PM (2007). Maternal Obesity and Risk of Gestational Diabetes Mellitus. Daibetes Care.

[10] O’Brien TE, Ray JG, Chan WS (2003). Maternal Body Mass Index and the Risk of Preeclampsia: a Systematic Overview. Epidemiology.

[11] Owens LA, O’Sullivan E, Kirwan B, Avalos G, Gaffney G, Dunne F (2010). ATLANTIC DIP: the impact of obesity on pregnancy outcome in glucose-tolerant women. Diabetes Care.

[12] Raatikainen K, Heiskanen N, Heinonen S (2006). Transition From Overweight to Obesity Worsens Pregnancy Outcome in a BMI-Dependent Manner. Obesity (Silver Spring).

[13] Rasmussen SA, Chu SY, Kim SY, Schmid CH, Lau J (2008). Maternal obesity and risk of neural tube defects: a metaanalysis. Am J Obstet Gynecol.

[14] Sebire NJ, Jolly M, Harris JP, Wadsworth J, Joffe M, Beard RW (2001). Maternal obesity and pregnancy outcome: a study of 287,213 pregnancies in London. Int J Obes Relat Metab Disord.

[15] Stothard KJ, Tennant PW, Bell R, Rankin J (2009). Maternal Overweight and Obesity and the Risk of Congenital Anomalies: a Systematic Review and Meta-Analysis. JAMA.

[16] Crozier SR, Inskip HM, Godfrey KM, Cooper C, Harvey NC, Cole ZA (2010). Weight gain in pregnancy and childhood body composition: findings from the Southampton Women’s Survey. Am J Clin Nutr.

[17] Pirkola J, Pouta A, Bloigu A, Hartikainen AL, Laitinen J, Jarvelin MR (2010). Risks of overweight and abdominal obesity at age 16 years associated with prenatal exposures to maternal prepregnancy overweight and gestational diabetes mellitus. Diabetes Care.

[18] Anderson AK, McDougald DM, Steiner-Asiedu M (2010). Dietary Trans Fatty Acid Intake and Maternal and Infant Adiposity. Eur J Clin Nutr.

[19] Bayol SA, Simbi BH, Fowkes RC, Stickland NC (2010). A Maternal “Junk Food” Diet in Pregnancy and Lactation Promotes Nonalcoholic Fatty Liver Disease in Rat Offspring. Endocrinology.

[20] Jones AP, Friedman MI (1982). Obesity and Adipocyte Abnormalities in Offspring of Rats Undernourished During Pregnancy. Science.

[21] Laitinen J, Jaaskelainen A, Hartikainen AL, Sovio U, Vaarasmaki M, Pouta A (2012). Maternal weight gain during the first half of pregnancy and offspring obesity at 16 years: a prospective cohort study. BJOG.

[22] Robinson T, Callister M, Jankoski T (2008). Portrayal of body weight on children’s television sitcoms: a content analysis. Body Image.

[23] Simmons SF, Keeler E, Zhuo X, Hickey KA, Sato HW, Schnelle JF (2008). Prevention of unintentional weight loss in nursing home residents: a controlled trial of feeding assistance. J Am Geriatr Soc.

[24] Carmichael SL, Yang W, Feldkamp ML, Munger RG, Siega-Riz AM, Botto LD, Shaw G, National Birth Defects Prevention Study (2012). Reduced Risks of Neural Tube Defects and Orofacial Clefts With Higher Diet Quality. Arch Pediatr Adolesc Med.

[25] Jensen CD, Block G, Buffler P, Ma X, Selvin S, Month S (2012). Maternal Dietary Risk Factors in Childhood Acute Lymphoblastic Leukemia. Cancer Causes and Control.

[26] Musselman JRB, Jurek AM, Johnson KJ, Linabery AM, Robinson LL, Shu X-O, Ross JA (2010). Maternal Dietary Patterns During Early Pregnancy and the Odds of Childhood Germ Cell Tumors: a Children’s Oncology Group Study. Am J Epidemiol.

[27] Orjuela MA, Titievsky L, Liu X, Ramirez-Ortiz M, Ponce-Castaneda V, Lecona E, Molina E, Beaverson K, Abramson DH, Mueller NE (2005). Fruit and Vegetable Intake During Pregnancy and Risk for Development of Sporadic Retinoblastoma. Cancer Epidemiol Biomarkers Prev.

[28] Petridou E, Ntouvelis E, Dessypris N, Terzidis A, Trichopoulos D, Childhood H-OG (2005). Maternal diet and acute lymphoblastic leukemia in young children. Cancer Epidemiol Biomarkers Prev.

[29] Brekke HK, Ludvigsson J (2010). Daily vegetable intake during pregnancy negatively associated to islet autoimmunity in the offspring–the ABIS study. Pediatr Diabetes.

[30] Chatzi L, Torrent M, Romieu I, Garcia-Esteban R, Ferrer C, Vioque J (2008). Mediterranean diet in pregnancy is protective for wheeze and atopy in childhood. Thorax.

[31] Aagaard-Tillery KM, Grove K, Bishop J, Ke X, Fu Q, McKnight R (2008). Developmental origins of disease and determinants of chromatin structure: maternal diet modifies the primate fetal epigenome. J Mol Endocrinol.

[32] Bray GA (2003). Risks of obesity. [Review]. Endocrinol Metab Clin North Am.

[33] Centers for Disease C. Prevention (2010). State-specific trends in fruit and vegetable consumption among adults — United States, 2000-2009. MMWR Morb Mortal Wkly Rep.

[34] Ervin RB (2011). Healthy Eating Index–2005 total and component scores for adults aged 20 and over: National Health and Nutrition Examination Survey, 2003-2004. Natl Health Stat Rep.

[35] Guenther PM, Dodd KW, Reedy J, Krebs-Smith SM (2006). Most Americans eat much less than recommended amounts of fruits and vegetables. J Am Diet Assoc.

[36] Guenther PM, Juan WY, Reedy J, Britten P, Lino M, Carlson A (2008). Diet quality of Americans in 1994-1996 and 2001-2002 as measured by the Healthy Eating Index-2005. FASEB J.

[37] Guenther P, Juan W, Lino M, Carlson A, Hiza H, Krebs-Smith S (2008). Diet quality of low-income and higher income Americans in 2003-04 as measured by the Healthy Eating Index-2005.

[38] Anderson AS (2001). Symposium on ‘Nutritional Adaptation to Pregnancy and Lactation’. Pregnancy As a Time for Dietary Change?. Proc Nutr Soc.

[39] Franz M, VanWormer J, Crain A, Boucher J, Histon T, Caplan W (2007). Weight-loss outcomes: a systematic review and meta-analysis of weight-loss clinical trials with a minimum 1-year follow-up. J Am Diet Assoc.

[40] US Department of Health and Human Services, National Heart Lung and Blood Institute (2013). Lifestyle Interventions to Reduce Cardiovascular Risk: Systematic Evidence Review from the Lifestyle Work Group.

[41] US Department of Health and Human Services, National Heart Lung and Blood Institute (2014). Guidelines (2013) for managing overweight and obesity in adults. Preface to the Expert Panel Report (comprehensive version which includes systematic evidence review, evidence statements, and recommendations). Obesity (Silver Spring).

[42] Lowe MR, Butryn ML (2007). Hedonic hunger: a new dimension of appetite?. Physiol Behav.

[43] Lutter M, Nestler EJ (2009). Homeostatic and hedonic signals interact in the regulation of food intake. J Nutr.

[44] Stice E, Spoor S, Bohon C, Veldhuizen MG, Small DM (2008). Relation of reward from food intake and anticipated food intake to obesity: a functional magnetic resonance imaging study. J Abnorm Psychol.

[45] Demos KE, Heatherton TF, Kelley WM (2012). Individual differences in nucleus accumbens activity to food and sexual images predict weight gain and sexual behavior. J Neurosci.

[46] Epstein LH, Leddy JJ, Temple JL, Faith MS (2007). Food reinforcement and eating: a multilevel analysis. Psychol Bull.

[47] Lietti CV, Murray MM, Hudry J, le Coutre J, Toepel U (2012). The Role of Energetic Value in Dynamic rain Response Adaptation During Repeated Food Image Viewing. Appetite.

[48] Stoeckel LE, Weller RE, Cook EW, Twieg DB, Knowlton RC, Cox JE (2008). Widespread Reward-System Activation in Obese Women in Response to Pictures of High-Calorie Foods. Neuroimage.

[49] Toepel U, Knebel JF, Hudry J, le Coutre J, Murray MM (2009). The Brain Tracks the Energetic Value in Food Images. Neueroimage.

[50] Gearhardt AN, Davis C, Kuschner R, Brownell KD (2011). The addiction potential of hyperpalatable foods. [Review]. Curr Drug Abuse Rev.

[51] Gearhardt AN, Yokum S, Orr PT, Stice E, Corbin WR, Brownell KD (2011). Neural correlates of food addiction. Arch Gen Psychiatry.

[52] Berridge KC, Kringelbach ML (2008). Affective Neuroscience of Pleasure: Reward in Humans and Animals. Psychopharmacology (Berl).

[53] Drewnowski A, Krahn DD, Demitrack MA, Nairn K, Gosnell BA (1992). Taste Responses and Preferences for Sweet High-Fat Foods: Evidence for Opioid Involvement. Physiol Behav.

[54] Green SM, Blundell JE (1996). Effect of Fat- and Sucrose-Containing Foods on the Size of Eating Episodes and Energy Intake in Lean Dietary Restrained and Unrestrained Females: Potential for Causing Overconsumption. Eur J Clin Nutr.

[55] Kessler D (2009). The end of overeating.

[56] US Department of Agriculture, Agricultural Research Service (2005). What we eat in America, NHANES 2001-2002: usual nutrient intakes from food compared to dietary reference intakes.

[57] McGuire S (2016). Scientific Report of the 2015 Dietary Guidelines Advisory Committee. Adv Nutr.

[58] Fulgoni VL, Keast DR, Drewnowski A (2009). Development and Validation of the Nutrient-Rich Foods Index: a Tool to Measure Nutritional Quality of Foods. J Nutr.

[59] U.S.Department of Agriculture USDoHaHS (2010). Dietary Guidelines for Americans, 2010.

[60] Beaver J, Lawrence A, van Ditzhuijzen J, Davis M, Woods A, Calder A (2006). Individual differences in reward drive predict neural responses to images of food. J Neurosci.

[61] Stice E, Yokum S, Bohon C, Marti N, Smolen A (2010). Reward circuitry responsivity to food predicts future increases in body mass: moderating effects of DRD2 and DRD4. Neuroimage.

[62] Rothemund Y, Preuschhof C, Bohner G, Bauknecht HC, Klingebiel R, Flor H, Klapp BF (2007). Differential Activation of the Dorsal Striatum by High-Calorie Visual Food Stimuli in Obese Individuals. Neuroimage.

[63] Cohen DA, Sturm R, Scott M, Farley TA, Bluthenthal R (2010). Not Enough Fruit and Vegetables or Too Many Cookies, Candies, Salty Snacks, and Soft Drinks?. Public Health Rep.

[64] Burger KS, Stice E (2011). Variability in reward responsivity and obesity: evidence from brain imaging studies. Curr Drug Abuse Rev.

[65] Robinson TE, Berridge KC (2000). The psychology and neurobiology of addiction: an incentive-sensitization view. Addiction.

[66] Alsio J, Olszewski PK, Levine AS, Schioth HB (2012). Feed-Forward Mechanisms: Addiction-Like Behavioral and Molecular Adaptations in Overeating. Front Neuroendocrinol.

[67] Bobroff EM, Kissileff HR (1986). Effects of Changes in Palatability on Food Intake and the Cumulative Food Intake Curve in Man. Appetite.

[68] Clark EN, Dewey AM, Temple JL (2010). Effects of daily snack food intake on food reinforcement depend on body mass index and energy density. Am J Clin Nutr.

[69] Temple JL, Bulkley AM, Badawy RL, Krause N, McCann S, Epstein LH (2009). Differential effects of daily snack food intake on the reinforcing value of food in obese and nonobese women. Am J Clin Nutr.

[70] Teegarden SL, Scott AN, Bale TL (2009). Early life exposure to a high fat diet promotes long-term changes in dietary preferences and central reward signaling. Neuroscience.

[71] Burger KS, Stice E (2012). Frequent Ice Cream Consumption Is Associated With Reduced Striatal Response to Receipt of an Ice Cream-Based Milkshake. Am J Clin Nutr.

[72] Clark EN, Dewey AM, Temple JL (2010). Effects of daily snack food intake on food reinforcement depend on body mass index and energy density. Am J Clin Nutr.

[73] Hetherington MM, Pirie LM, Nabb S (2002). Stimulus satiation: effects of repeated exposure to foods on pleasantness and intake. Appetite.

[74] Temple JL, Chappel A, Shalik J, Volcy S, Epstein LH (2008). Daily consumption of individual snack foods decreases their reinforcing value. Eat Behav.

[75] Tey SL, Brown RC, Gray AR, Chisholm AW, Delahunty CM (2012). Long-term consumption of high energy-dense snack foods on sensory-specific satiety and intake. Am J Clin Nutr.

[76] Guerrieri R, Nederkoorn C, Jansen A (2007). How impulsiveness and variety influence food intake in a sample of healthy women. Appetite.

[77] Guerrieri R, Nederkoorn C, Stankiewicz K, Alberts H, Geschwind N, Martijn C (2007). The influence of trait and induced state impulsivity on food intake in normal-weight healthy women. Appetite.

[78] Bonato DP, Boland FJ (1983). Delay of gratification in obese children. Addict Behav.

[79] Nederkoorn C, Braet C, Van Eijs Y, Tanghe A, Jansen A (2006). Why obese children cannot resist food: the role of impulsivity. Eat Behav.

[80] Nederkoorn C, Smulders FT, Havermans RC, Roefs A, Jansen A (2006). Impulsivity in obese women. Appetite.

[81] Appelhans BMWK, Pagoto SL, Schneider KL, Whited MC, Liebman R (2011). Inhibiting food reward: delay discounting, food reward sensitivity, and palatable food intake in overweight and obese women. Obesity (Silver Spring).

[82] Rollins BY, Dearing KK, Epstein LH (2010). Delay discounting moderates the effect of food reinforcement on energy intake among non-obese women. Appetite.

[83] Epstein LH, Lin H, Carr KA, Fletcher KD (2012). Food reinforcement and obesity. Psychological moderators. Appetite.

[84] Goldfield GS, Lumb A (2009). Effects of dietary restraint and body mass index on the relative reinforcing value of snack food. Eat Disord.

[85] Lowe MR (2003). Self-regulation of energy intake in the prevention and treatment of obesity: is it feasible?. Obes Res.

[86] Mumford SL, Siega-Riz AM, Herring A, Evenson KR (2008). Dietary restraint and gestational weight gain. J Am Diet Assoc.

[87] Kerr KL, Avery JA, Barcalow JC, Moseman SE, Bodurka J, Bellgowan PS (2014). Trait impulsivity is related to ventral ACC and amygdala activity during primary reward anticipation. Soc Cogn Affect Neurosci.

[88] Batterink L, Yokum S, Stice E (2010). Body mass correlates inversely with inhibitory control in response to food among adolescent girls: an fMRI study. Neuroimage.

[89] Kishinevsky FI, Cox JE, Murdaugh DL, Stoeckel LE, Cook EW, Weller RE (2012). fMRI reactivity on a delay discounting task predicts weight gain in obese women. Appetite.

[90] Yokum S, Ng J, Stice E (2011). Attentional bias to food images associated with elevated weight and future weight gain: an fMRI study. Obesity (Silver Spring).

[91] Subar AF, Kirkpatrick SI, Mittl B, Zimmerman TP, Thompson FE, Bingley C (2012). The Automated Self-Administered 24-hour dietary recall (ASA24): a resource for researchers, clinicians, and educators from the National Cancer Institute. J Acad Nutr Diet.

[92] Kirkpatrick SI, Subar AF, Douglass D, Zimmerman TP, Thompson FE, Kahle LL (2014). Performance of the Automated Self-Administered 24-hour Recall relative to a measure of true intakes and to an interviewer-administered 24-h recall. Am J Clin Nutr.

[93] Fein SB, Labiner-Wolfe J, Shealy KR, Li R, Chen J, Grummer-Strawn LM (2008). Infant Feeding Practices Study II: study methods. Pediatrics.

[94] Sharma S, Kolahdooz F, Butler L, Budd N, Rushovich B, Mukhina GL (2013). Assessing dietary intake among infants and toddlers 0-24 months of age in Baltimore, Maryland, USA. Nutr J.

[95] Johnson L, Llewellyn CH, van Jaarsveld CH, Cole TJ, Wardle J (2011). Genetic and environmental influences on infant growth: prospective analysis of the Gemini twin birth cohort. PLoS One.

[96] Stifter CA, Anzman-Frasca S, Birch LL, Voegtline K (2011). Parent use of food to soothe infant/toddler distress and child weight status. An exploratory study. Appetite.

[97] Lowe MR, Butryn ML, Didie ER, Annunziato RA, Thomas JG, Crerand CE (2009). The Power of Food Scale. A new measure of the psychological influence of the food environment. Appetite.

[98] Cappelleri JC, Bushmakin AG, Gerber RA, Leidy NK, Sexton CC, Karlsson J (2009). Evaluating the Power of Food Scale in obese subjects and a general sample of individuals: development and measurement properties. Int J Obes (Lond).

[99] Flint AJ, Gearhardt AN, Corbin WR, Brownell KD, Field AE, Rimm EB (2014). Food-addiction scale measurement in 2 cohorts of middle-aged and older women. Am J Clin Nutr.

[100] Epstein LH, Salvy SJ, Carr KA, Dearing KK, Bickel WK (2010). Food reinforcement, delay discounting and obesity. Physiol Behav.

[101] Griffiths RR, Troisi JR, Silverman K (1993). Mumford GK. Multiple-choice procedure: an efficient approach for investigating drug reinforcement in humans. Behav Pharmacol.

[102] Van Strien T, Frijters JER, Bergers JPA, Defares PB (1986). The Dutch Eating Behavior Questionnaire (DEBQ) for assessment of restrained, emotional, and external eating. Int J Eat Disord.

[103] Levesque CS, Williams GC, Elliot D, Pickering MA, Bodenhamer B, Finley PJ (2007). Validating the theoretical structure of the Treatment Self-Regulation Questionnaire (TSRQ) across three different health behaviors. Health Educ Res.

[104] Lohse B, Satter E, Horacek T, Gebreselassie T, Oakland MJ (2007). Measuring eating competence: psychometric properties and validity of the ecSatter Inventory. J Nutr Educ Behav.

[105] Lim J, Wood A, Green BG (2009). Derivation and evaluation of a labeled hedonic scale. Chem Senses.

[106] Hoerger M, Quirk SW, Weed NC (2011). Development and validation of the Delaying Gratification Inventory. Psychol Assess.

[107] Spinella M (2007). Normative data and a short form of the Barratt Impulsiveness Scale. Int J Neurosci.

[108] Fulkerson JA, Nelson MC, Lytle L, Moe S, Heitzler C, Pasch KE (2008). The validation of a home food inventory. Int J Behav Nutr Phys Act.

[109] Stunkard AJ, Messick S (1985). The three-factor eating questionnaire to measure dietary restraint, disinhibition and hunger. J Psychosom Res.

[110] Evenson KR, Wen F (2010). National trends in self-reported physical activity and sedentary behaviors among pregnant women: NHANES 1999-2006. Prev Med.

[111] Cohen S, Kamarck T, Mermelstein R (1983). A global measure of perceived stress. J Health Soc Behav.

[112] Cohen S, Williamson GM (1988). Perceived Stress in a Probability Sample of the United States. Social Psychology of Health.

[113] Hoch CC, Buysse DJ, Reynolds CF (1989). Sleep and depression in late life. Clin Geriatr Med.

[114] Cox JL, Holden JM, Sagovsky R (1987). Detection of postnatal depression. Development of the 10-item Edinburgh Postnatal Depression Scale. Br J Psychiatry.

[115] Guenther PM, Kirkpatrick SI, Reedy J, Krebs-Smith SM, Buckman DW, Dodd KW (2014). The Healthy Eating Index-2010 is a valid and reliable measure of diet quality according to the 2010 Dietary Guidelines for Americans. J Nutr.

